# Tree rings as a proxy for seasonal precipitation variability and Early Neolithic settlement dynamics in Bavaria, Germany

**DOI:** 10.1371/journal.pone.0210438

**Published:** 2019-01-30

**Authors:** Joachim Pechtl, Alexander Land

**Affiliations:** 1 kelten römer museum manching, Manching, Germany; 2 Institute of Botany (210a), University of Hohenheim, Stuttgart, Germany; 3 University of Applied Forest Sciences, Rottenburg am Neckar, Germany; Woods Hole Oceanographic Institution, UNITED STATES

## Abstract

Studying the dynamic of Neolithic settlement on a local scale and its connection to climate variability is often difficult due to missing on-site climate reconstructions from natural archives. Here we bring together archaeological settlement data and a regional climate reconstruction from precipitation-sensitive trees. Both archives hold information about regional settlement dynamics and hydroclimate variability spanning the time of the first farming communities, the so called Linearbandkeramik (LBK) in Bavaria, Germany. Precipitation-sensitive tree-ring series from subfossil oak are used to develop a spring-summer precipitation reconstruction (5700–4800 B.C.E.) representative for southern Germany. Early Neolithic settlement data from Bavaria, mainly for the duration of the LBK settlement activities, are critically evaluated and compared to this unique regional hydroclimate reconstruction as well as to reconstructions of Greenland temperature, summer sea surface temperature, delta ^18^O and global solar irradiance to investigate the potential impact of climate on Neolithic settlers and their settlement dynamic during the LBK. Our hydroclimate reconstruction demonstrates an extraordinarily high frequency of severe dry and wet spring-summer seasons during the entire LBK, with particularly high year-to-year variability from 5400 to 5101 B.C.E. and with lower fluctuations until 4801 B.C.E. A significant influence of regional climate on the dynamic of the LBK is possible (e.g. around 4960 B.C.E.), but should be interpreted very carefully due to asynchronous trends in settlement dynamics. Thus, we conclude that even when a climate proxy such as tree rings that has excellent spatio-temporal resolution is available, it remains difficult to establish potential connections between the settlement dynamic of the LBK and climate variability.

## Introduction

In vast parts of Central Europe the first farming communities belong to a cultural phenomenon referred to as Linearbandkeramik (LBK) in archaeological terminology. Due to the enormous cultural importance of the rise of this new way of life which defines the beginning of the Neolithic period, the LBK has gained major attention in archaeological research [1–6]. Domestication of plants and animals as well as the development of many technical and cultural features related to an agrarian economy took place in the Fertile Crescent over a long period, giving rise to proper Neolithic cultures in the 9th– 8th millennium B.C.E. This new lifestyle spread from its place of origin through Anatolia and the Balkan Peninsula, and in the early 6th millennium B.C.E. finally reached the Carpathian Basin. Based on these roots, the LBK most likely developed in the north-western part of the Carpathian Basin during the 56th century B.C.E. [3,7]. Shortly thereafter, settlements of this specific culture were widespread from France in the west to Ukraine in the east. While the LBK itself ended at the turn of the 6th millennium B.C.E., it was followed by a group of different, but closely related cultures of the Middle Neolithic that continued many old traditions from the 60th century B.C.E. until the 45th century B.C.E. In northwest of Bavaria, the Middle Neolithic is divided in the subsequent cultural phases of Hinkelstein (HST; ca. 4904–4850 B.C.E.), Großgartach (GG; ca. 4850–4700 B.C.E.) and Rössen (RÖ; ca. 4700–4450 B.C.E.). Simultaneously, in southeast Bavaria, the successive phases of Stichbandkeramik (SBK; ca. 4950–4750 B.C.E.) and Oberlauterbach (OLB; ca. 4750–4450 B.C.E.) were present, together referred to as Südostbayerisches Mittelneolithikum (SOB) [8,9]. The LBK is characterised by a rapid expansion, a strong colonialistic tendency, an enormous distribution area and a massive increase in population density compared to previous Mesolithic hunter-gatherer societies [3,10–12]. Hence, it is considered an exceptionally effective cultural model in general [13]. This successful development is based on the foundation of the LBK’s culture-specific economic system, the basic features of which are becoming increasingly clear thanks to research results from various disciplines. Archaeological findings include the remains of both cultivated plants and collected wild plants [6,14] as well as technical equipment for the cultivation, harvesting, storage and processing of plants such as cereals [15]. Furthermore, bones, mainly of domestic animals (cattle, pig, sheep, goat and dog), are found accompanied by rare finds of bones of game [6,15,16]. As kill off patterns and analyses of lipid residues in ceramic shards show, the primary focus of husbandry was meat production while milk played a comparatively minor roll [17,18]. Palynological analyses reveal the impact of crop production and animal husbandry on the vegetation and document the cultivation of cereals [15,19]. Isotope analyses on human skeletal material indicate a largely plant-based diet [20,21]. As a whole, the LBK economic system largely depended on agriculture, especially on cultivated cereals. Most important is that agriculture was based on only four crops (emmer, einkorn, pea and lentil), and that the agricultural system seems to have been quite simple and labour-saving [22]. For example, although the technology of artificial wells was available at that time [23], there is no evidence of irrigation. The conservative perpetuation of this economic system over most parts of the LBK-territory for centuries and the obvious refusal of adaptive strategies is remarkable. For instance, increased hunting or intensification of pig breeding to cope with difficult times are options that were rarely utilized [24].

Temporally, the sequence from later Mesolithic to Early and Middle Neolithic coincides with the Atlantic period which is also referred to as the Holocene Climatic Optimum (7000–3800 B.C.E.) [25]. Generally speaking and based on proxy-data with centennial or decadal resolution, during this phase the palaeoclimatic conditions were reasonably stable, somewhat warmer and more humid than in modern times [26]. Hence, during the 20th century the archaeological discourse typically presumed quite favourable conditions for early agrarian societies in Middle Europe and assumed that any climatic impacts on cultural development were negligible [15]. The recent appearance of highly resolved palaeoclimatic proxy-data during the last decades in combination with the heated political debate about recent climatic change have led to the generation of explicitly climate-deterministic models in Neolithic archaeology, which even postulate a disastrous end to the LBK [27].

**Fig 1:**
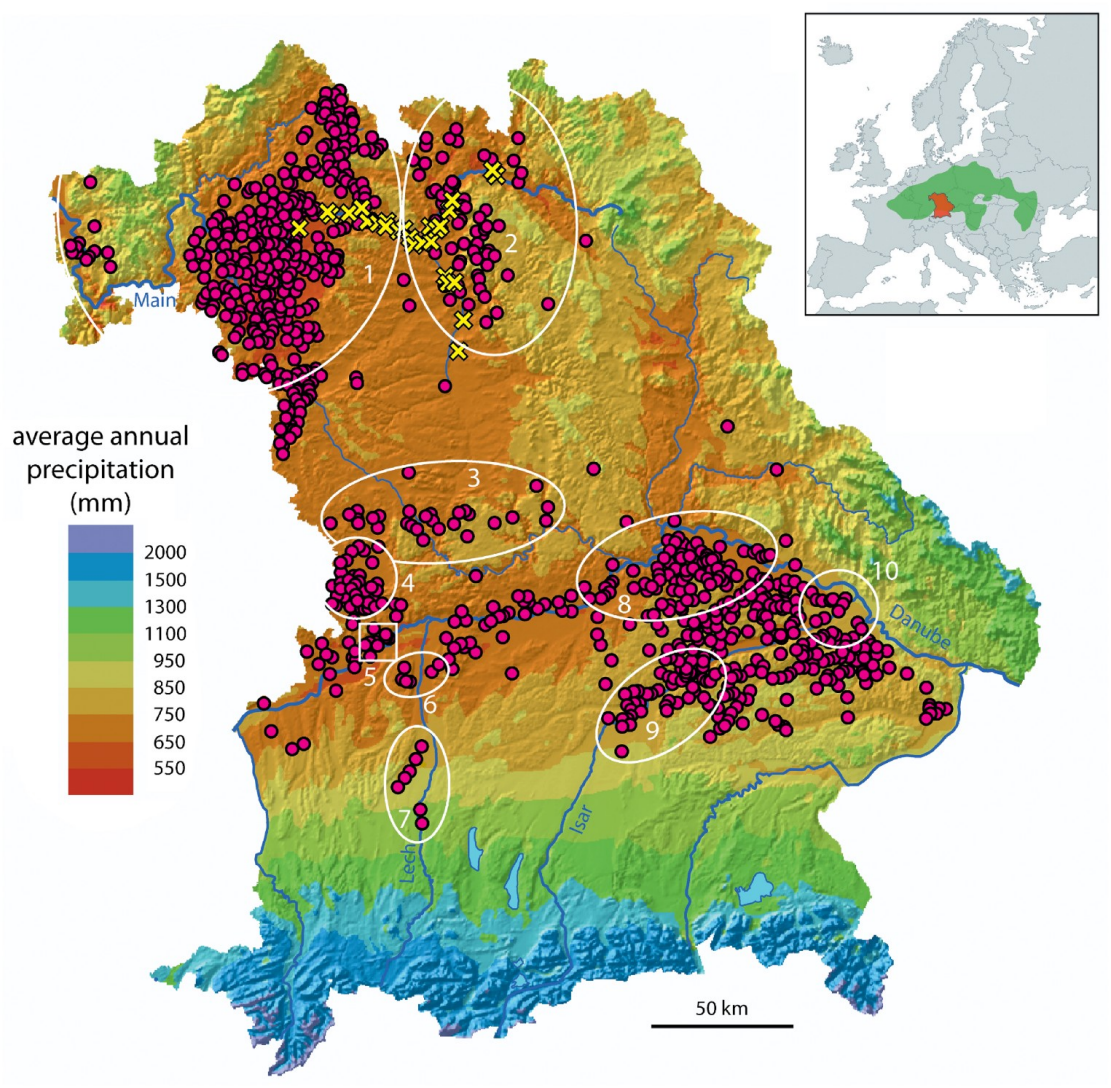
Distribution of LBK settlements (red dots) in relation to recent average annual precipitation in Bavaria. Regions analyzed concerning the settlement dynamic during LBK and early Middle Neolithic: 1 Lower Franconia, 2 Upper Franconia, 3 south-central Franconia, 4 Ries, 5 upper Danube/Höchstädt region, 6 Lech estuary, 7 middle Lech, 8 Danube/Regensburg region, 9 middle Isar, 10 Isar estuary. Sites where the analyzed oaks were found are indicated by yellow crosses. The small map indicates the entire distribution of the LBK in Europe (green) and the position of Bavaria (red).

Actually, it is a well-established fact that during the LBK and the early Middle Neolithic the selection criteria for settlement sites were quite strict in order to meet very high agrarian needs for the cultivation of crops. Fertile soils, high temperatures correlating to a long vegetation period and low precipitation have been identified as particularly crucial requirements. Additionally, a flood-free position of the settlement in addition to good access to freshwater at natural sources or artificial wells in direct vicinity were important [28]. In Bavaria for instance, this cherry picking strategy lead to insular settlement concentrations especially in the basins of the Main and Danube rivers, while extended regions were completely bare of Neolithic settlement, such as the Bavarian Forest or the Alpine Foreland (Fig 1). Apparently, successful agriculture was restricted to a narrow corridor of suitable natural and climatic conditions. Hence, an impact of spatial climatic variability on the LBK is probable, and it seems very likely that temporal climatic variability also affected the development of the LBK. Further specification is, however, still necessary to determine where and when such effects occurred, how climatic change actually influenced socio-economic systems and what kind of cultural reactions occurred. At best, this can be achieved in a joint palaeoclimatic and archaeological investigation. This paper presents data from both disciplines for the territory of Bavaria from the timespan 5700–4800 B.C.E.

## Materials and methods

### Archaeological sources of LBK settlement in Bavaria

Due to the solid construction of the houses, the frequent installation of large pits and the easily identified find material, the archaeological remains of the LBK are far more frequent than those of many other prehistoric cultures in Central Europe. The overall distribution of the LBK in Bavaria can therefore be reliably determined on the basis of the data of the Bavarian State Office for the Preservation of Historical Monuments (Fig 1). While an overall evaluation for Bavaria has not been conducted to date, there are studies for some sub-regions from which the number and relative chronology of the occupancy time of the known settlements can be determined (e.g. [28,29–32]). Valuable unpublished data of this type was also provided for the Ries by Anna-Leena Fischer, for Lower Franconia by Jessica Siller and for Upper Franconia by Alexa Dürr. Such studies are predominantly based on surface findings.

Substantially detailed data including information on settlement sizes and numbers of ground plans for individual localities can be found in a variety of preliminary excavation reports and final publications (extensive bibliography in: [33,34]). In fact, heavy erosion that results in poorly preserved sites sometimes limits the amounts of information that can be retrieved from the investigated sites. For example, extensive excavations in Niederpöring revealed numerous LBK settlement features, but not a single ground plan was preserved due to large-scale erosion [35], while in nearby Otzing dozens of ground plans are present in a comparable area [13]. Even on relatively well-preserved settlement sites like nearby Stephansposching, the loss of soil since the Neolithic is estimated to around 50 cm [36].

To date, the only intensive study on the history of LBK settlement in Bavaria took place around Höchstädt a. d. Donau (Fig 1 no. 5). There, excavation results were combined with intensive archaeological field walking, a geophysical survey and aerial photography to explore the settlement history of a mirco-region in detail [37].

While the validity of individual datasets on settlement-historical issues is thus partly insufficient, the sum of all data provides a comprehensive basis for such evaluations.

### Relative chronology in Bavaria

Chronological classification of Early Neolithic archaeological material is dependent predominantly on statistical analyses of the development of decorated ceramics, which allow for relative chronological models with a resolution down to human generations possible in some cases [37,38]. Four regional chronologies are suitable for relative dating within the major LBK settlement areas of Bavaria in Upper and Lower Franconia [29], in Middle Franconia [30], in south-western Bavaria [38] and in south-eastern Bavaria [33]. The relatively coarse-grained division in only five phases (I–V) and the related denomination as oldest, older, middle, younger and youngest LBK of the Meier-Arendt-system [29] are most suitable for interregional comparison. Also for early Middle Neolithic material suitable systems are at hand [9,39].

### Data for absolute LBK chronology

A huge body of ^14^C-data is available from sites throughout the entire distribution area of the LBK [40]. Although initially derived mainly from charcoal, modern ^14^C-analyses are primarily based on short-lived plant material or bone from settlement features as well as from burials. Up to now it has been fairly impossible to create a widely accepted absolute fine-chronology and to date typological phases precisely. This unsatisfactory state of knowledge is the result of a series of fluctuations in the ^14^C-calibration curve, the most notable of which is a plateau in the calibration curve covering virtually the entire Bandkeramik period (recent attempts of absolute dating: [9,41,42]. Dendrochronology also contributes some additional information. But firstly, findings of sufficiently preserved wood in LBK settlements are rare [23,43]. Secondly, the fitting of the determined series into the standard curve is sometimes problematic [44]. Thirdly, the temporal relation between wooden structures and ceramic finds is not entirely clear. In fact, there is a complete lack of dendrochronological dates for the LBK from Bavaria.

The available chronological data therefore enable an estimation of the beginning and the end and thus the entire duration of the LBK, but are insufficient for achieving an annually-resolved chronology. Only the transition from the oldest to the older LBK can still be roughly estimated.

### Constructing a chronological model for the Bavarian Early Neolithic

Creating a chronological model for the Bavarian Early Neolithic is a challenge. In a first step, the different regional relative chronologies have to be synchronised. This is done through a typological comparison of the spectra of decoration features on ceramics of the individual stages of the LBK and the early Middle Neolithic within Bavaria (methodology: [33,38]). Most valuable in this process is the correlation to well-established chronological systems of surrounding LBK-provinces, especially to Württemberg, Bohemia and Lower Austria [2,3,9,38,39,41,45].

Based on the construction sequence achieved in Stephansposching, in a second step, at least for the stages of the older to younger LBK, the number of house generations can be estimated, which is used as a measure of the relative duration of individual stages [33].

If, in a third step, the absolute dates of the total duration of the LBK and the presumed beginning of the older LBK in Bavaria are included, then this information can be used to form a chronological model of the absolute dating and duration of individual ceramic-defined stages in the different regions.

In fact, the biggest uncertainties of this model are due to the integration of the absolute dates, as both the beginning and the end of the LBK are discussed in contradictory terms. For the beginning of the oldest LBK in southern Germany, Strien [3] brought very early dates in the discussion. These are in contrast to the previous chronology models for the oldest LBK in Bavaria [5,46] and the renewed ^14^C-dating of the important findings from Schwanfeld underpins a more recent time approach [47]. Therefore the beginning of the oldest LBK in Franconia is accepted around 5500 B.C.E. and in southern Bavaria around 5400 B.C.E. Link [45] convincingly argues for a continuous development from LBK to early SBK in the upper Elbe region and dates this to the time around 5000 ±50 B.C.E., while Riedhammer [9] proposes an end of the LBK around 5050 B.C.E. for southern Bavaria, followed by a hiatus and a start of early SBK in the second half of the 50th century B.C.E. In contrast, the beginning of HST in western Germany and Alsace is dated to the 52nd century B.C.E. by Jeunesse and Strien [48] although its close connection to early SBK is commonly accepted. In regards to Bavaria, it is very probable that some settlements and even some burial grounds in southern Bavaria were occupied or used continuously from the LBK until the early Middle Neolithic, and both cultures are connected by numerous typological similarities [33]. Therefore, in this paper, a continuous development from LBK to the Middle Neolithic in the early 50th century B.C.E. is assumed with the possibility of a certain temporal overlap of the ceramic styles.

**Fig 2:**
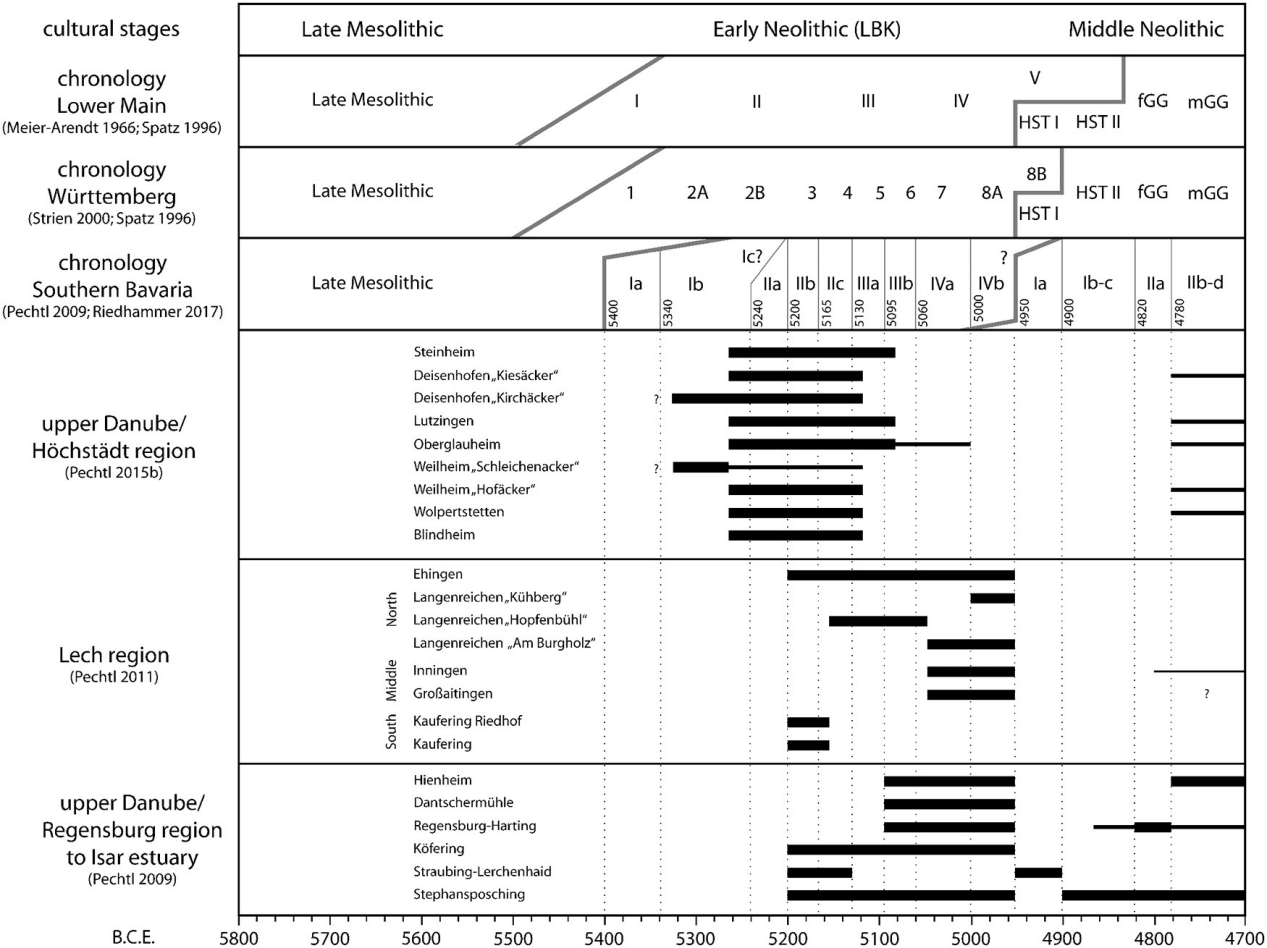
Occupancy of single settlement sites at the upper Bavarian Danube and at the Lech during the LBK and the early Middle Neolithic.

**Fig 3:**
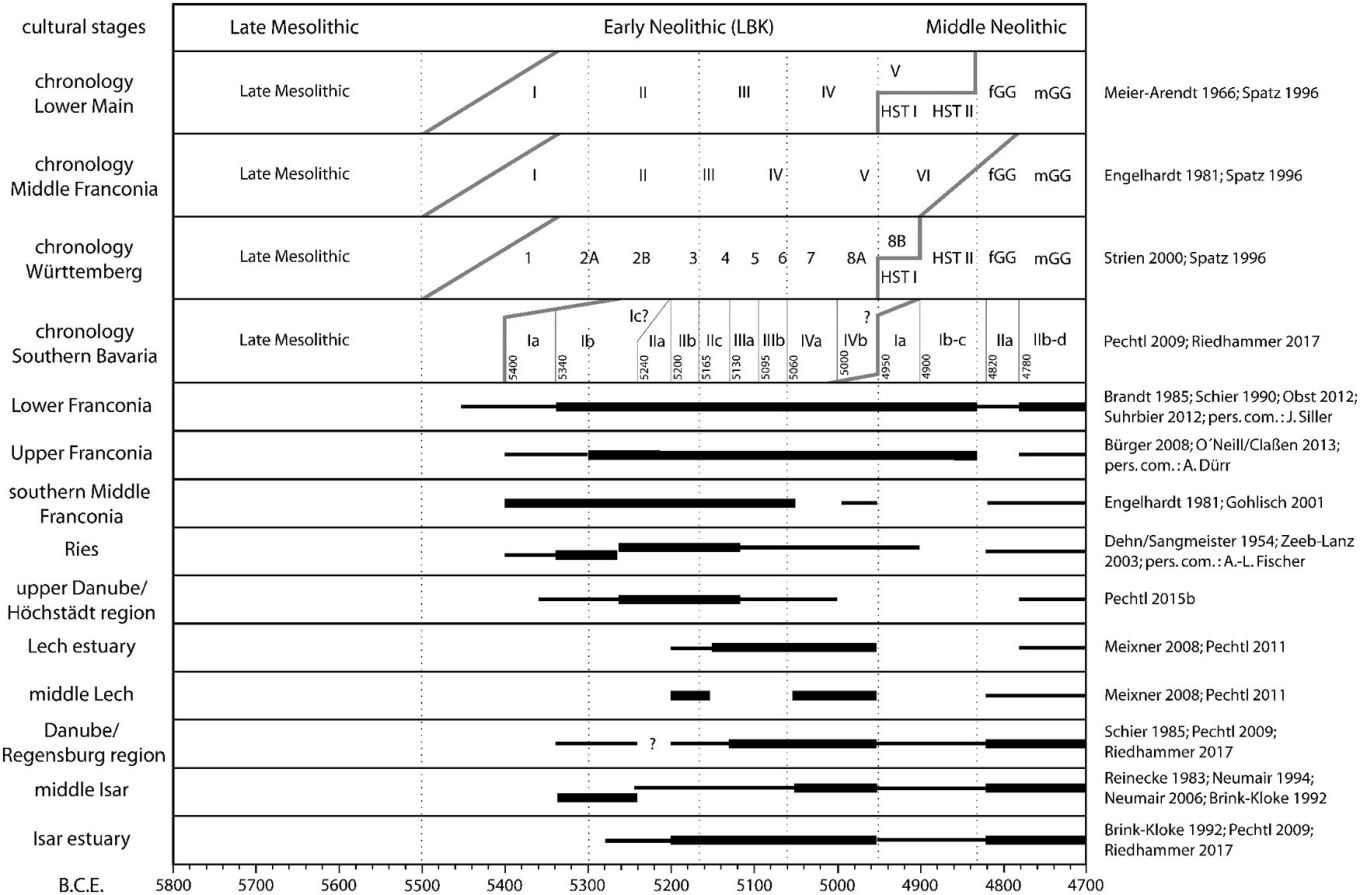
Comparison of the settlement dynamics during the LBK and the early Middle Neolithic in different settlement regions in Bavaria, Germany. Narrow lines indicate moderate settlement, thick lines indicate intensive settlement and lateral off-set equals spatial discontinuity of settlement.

Hence, the complete chronological system proposed here (Figs 2 and 3), including the correlation of different regional systems as well as the absolute dating of stylistic phases, is simply a model containing several compromises. At the moment, it is impossible to define an exact confidence interval for the absolute dating, but the deviation is estimated not to exceed a maximum of 100 years.

According to this chronological model, the five sub-phases defined by Meier-Arendt [29] are dated as follows: oldest LBK 5500–5300 B.C.E., older LBK 5300–5165 B.C.E., middle LBK 5165–5060 B.C.E., younger LBK 5060–4955 B.C.E. and the youngest LBK (which is not present in all regions and coincides with the early Middle Neolithic) 4955–4835 B.C.E.

### Generating a model of Bavarian Early Neolithic settlement

At best, studies on Early Neolithic settlement dynamics are based on the identification of settlement sites, the determination of size and duration of settlement activities as well as the number of houses on single sites and the comparison to other sites within a study area. To maximize the amount of information retrieved from a site, large scale excavation, geophysical survey and intense archaeological field walking are recommendable (e.g. [49]). In Bavaria just a single project of this type was carried out in the region around the modern town of Höchstädt in the uppermost part of the Bavarian Danube valley [37]. Although the investigation area only covers 10 km*10 km, nine LBK settlements are known, which were the object of intense research. Hence, in this micro-region the development of the settlement is known in detail (Fig 2).

If statements are to be made regarding a larger area, as done here regarding all of Bavaria, the settlement of various small regions must be presented in a comparable way despite the often different sources. For this purpose, the above-mentioned sources were evaluated as to whether and in which phases (according to the respective locally applicable relative chronology system) settlement in certain sub-regions is detectable. In addition, very sparse settlement areas are distinguished from areas where settlement activity regularly occurs. Evidence of settlement relocation is also taken into account. At least parts of all important LBK settlement regions of Bavaria were included according to the state of current research (Fig 1). After the synchronisation of the results for the individual sub-regions with the described chronological model, the development of LBK settlement across Bavaria can be compared and precisely dated (Fig 3).

The chart gives semi-quantitative information about the presence of settlement materials of the different chronological phases in the included regions. This enables the description of the settlement history of a region. Thus, not only the beginning and end of the settlement can be determined, but it is also possible to capture the dynamics of the settlement, including all changes or deviations from a continuous development. These include the increase or decrease in the number of households in a region as well as the resettlement or abandonment of an area or the relocation of settlement activities with the same number of households. A high settlement dynamic in this sense therefore means a strong temporal change, but not necessarily an increase in population. Since it is possible to deduce the human population from the number of simultaneous houses, the human population can be estimated.

### Tree-ring series of subfossil oaks

Fig 1 presents the geographical origin of the subfossil oak trunks used for the tree-ring based climate reconstruction. The tree-ring series used to reconstruct the precipitation variability in the LBK are part of the Hohenheim tree-ring archive [50,51]. The subfossil oaks were found in alluvial deposits (gravel pits) close to the River Main. The total ring width (TRW) was measured with a resolution of 1/100 mm and then dendrochronologically dated.

For this study a set of 354 precisely dated oak tree-ring series [52] (DOI: 10.5281/zenodo.1326474) from the Main region in Bavaria (southern Germany) have been used to develop a TRW chronology. The tree-ring series span from 5903 to 4451 B.C.E. This ~1,500-year period was chosen to ensure a sufficient temporal coverage of the LBK in southern Germany (5700–4800 B.C.E.). We would like to note that oak tree-ring series from the upper Danube could not be used for this study due to missing subfossil samples during the LBK.

### Standardization of TRW series and chronology construction

Standardization of the tree-ring series was performed using the software ARSTAN [53]. A flexible (70%) cubic smoothing spline with 50% frequency response cut-off was applied to each series to preserve high- to mid-frequency variability. After fitting the growth curves, annual indices were calculated as ratios from the fitted growth curves and the TRW series. Variance was adjusted [54] by taking into account the variable replication over time in the TRW set. A composite chronology was developed using the bi-weight robust mean method. To assess the signal strength of the constructed chronology, Expressed Population Signal (EPS) and inter-series correlation (RBAR) [55] were calculated to ensure sufficient sample replication over time. EPS and RBAR were calculated in 50-year windows with 25 years of overlap. In dendroclimatology it is widely accepted that an EPS close to one show a high signal strength of the TRW chronology.

### Climate-growth model: Calibration and verification

The here used oak trees are known to be rainfall sensitive in spring-summer season [56]. Climate-growth model calibration was performed over a 50-year (1879–1928) and verified over an independent period. Additionally, calibration was performed over the full period from 1879 to 1978. We used linear regression for the calibration and the coefficient of efficiency (CE) [57] while the verification. A CE value of >0 was assumed to indicate a robust reconstruction.

### The climate dynamics between 5700 and 4800 B.C.E.

#### Spring-summer precipitation variability inferred from tree rings

Radial growth (measured as total ring width, TRW) of oak trees (*Quercus robur* and *Q*. *petraea*) in central Europe is sensitive to the amount of precipitation during the vegetation period [58] or drought [59,60]. Thus, oak TRW serves as a precise indicator for seasonal precipitation totals/droughts and can be ideally used to reconstruct the hydroclimate variability in ancient times [61,62].

To reconstruct the hydroclimate variability, we used precipitation-sensitive subfossil oak trees from the upper Main region (Fig 1). Due to a relatively uniform rainfall distribution within the upper Main region, the oak trees reflect the regional hydroclimate as well as to a wider geographical extent, at least for the area of interest. The developed climate-growth model was applied to reconstruct the hydroclimate variability in the study region. The Mean Absolute Error (MAE) was used to evaluate average model performance of the developed climate-growth relationship [63,64].

The oak trees used here reflect a seasonal hydroclimatic signal and serve as a highly-resolved climate proxy for southern Germany. The presented reconstruction holds information about the precipitation totals between April 14 and July 18 (mid-spring to mid-summer). The applied model slightly underestimates the total amount of spring-summer rainfall in the case of singular, short, heavy rainfall events (exceeding 7.5 mm/day) (for detailed information see [56]).

The raw values of the hydroclimate reconstruction were transformed into z-scores corresponding to the number of standard deviations between the raw value and the mean of the distribution. A z-score of <-2 was defined as a severe drought and of >+2 as a severe wet accounting for less than 5% of the data points.

It is important to mention that hydroclimate variability on a low-frequency scale cannot be deduced from the tree-ring data due to the standardization procedure.

#### Paleoclimate variability inferred from other proxy data

We used estimations of Greenland temperature [65], summer sea surface temperature (sSST) of the northern North Atlantic [66], delta ^18^O ratios (reflecting air temperature) from southern Germany [67] and global solar irradiance [68] to investigate a possible paleoclimatic influence on the Early Neolithic settlement dynamics in Bavaria (Germany). The data reflect the situation in the arctic part of North America, in the North Atlantic and in southern Germany (Ammersee) close to the study area. These highly-resolved data reflect the evolution on decadal time scale and serve as a counterpart to the seasonally-resolved precipitation reconstruction from tree-ring data to study the climate impact on ancient society. To ensure a good comparison, we normalized (z-transformation) the above-mentioned data within the period 5700–4800 B.C.E.

The available spatial and temporal climate information of the data sets, especially of the high resolution hydroclimate reconstruction, offer the unique possibility to investigate the dynamic of the settlement development (renunciation and return) of the ancient LBK societies in regard to a changing climate in southern Germany.

## Results

### Calibration, verification statistics and model performance

**Fig 4:**
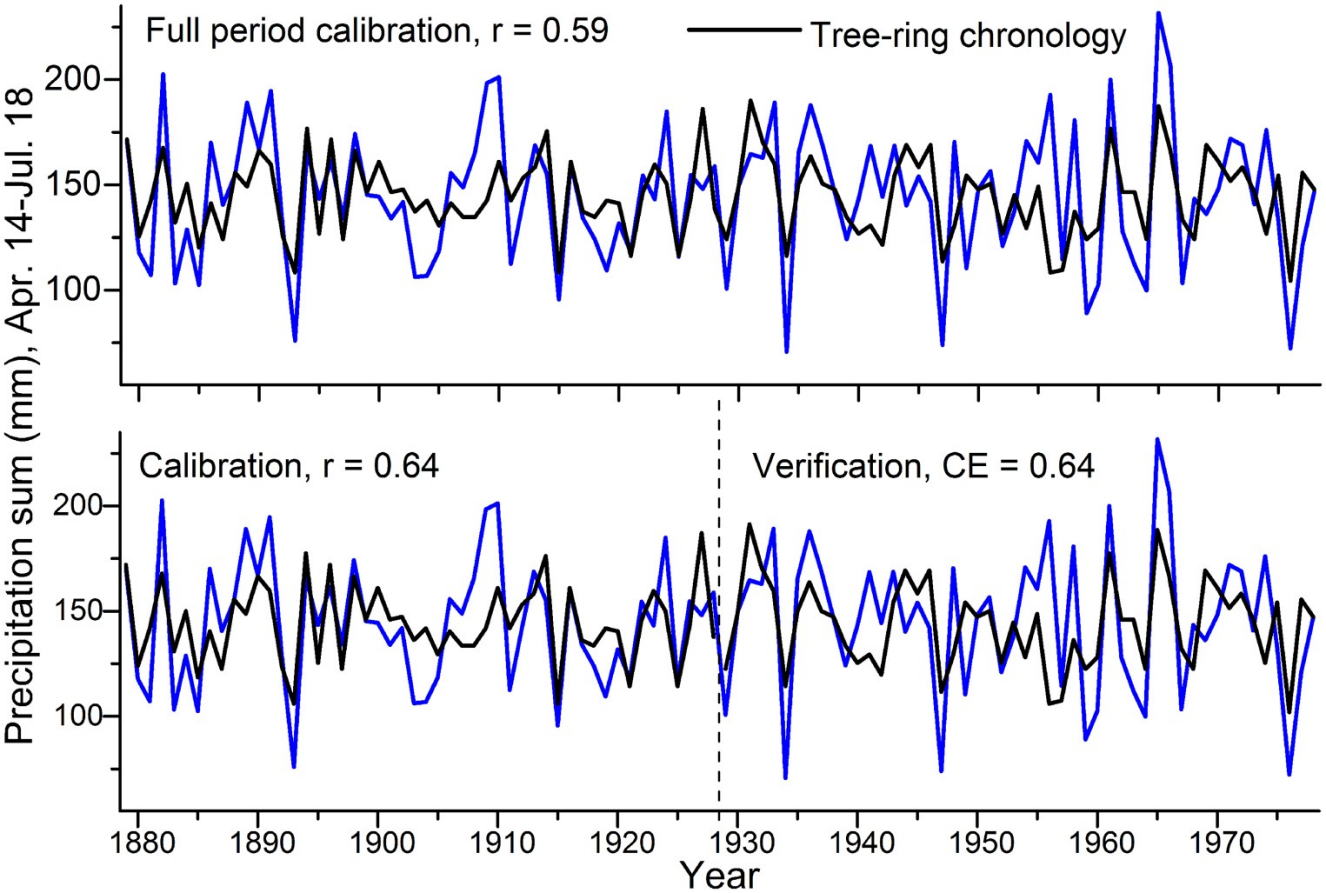
Results of the split period and full period calibration and verification of the climate-growth analysis.

Fig 4 shows the result of the calibration. Over the full period the correlation coefficient is 0.59 and for the 50-year split period is 0.64. The CE over the 50-year verification period is 0.64, indicating a robust reconstruction. Over the full period the TRW chronology explains 35% and over the split period 41% of precipitation variability. In seasons with very low rainfall (1893, 1934, 1947, 1976), TRW does not track extremely low precipitation rates adequately. Thus, the reconstructed spring-summer droughts during the LBK could be more intense than what TRW suggests.

### Replication and quality of the TRW chronology

**Fig 5:**
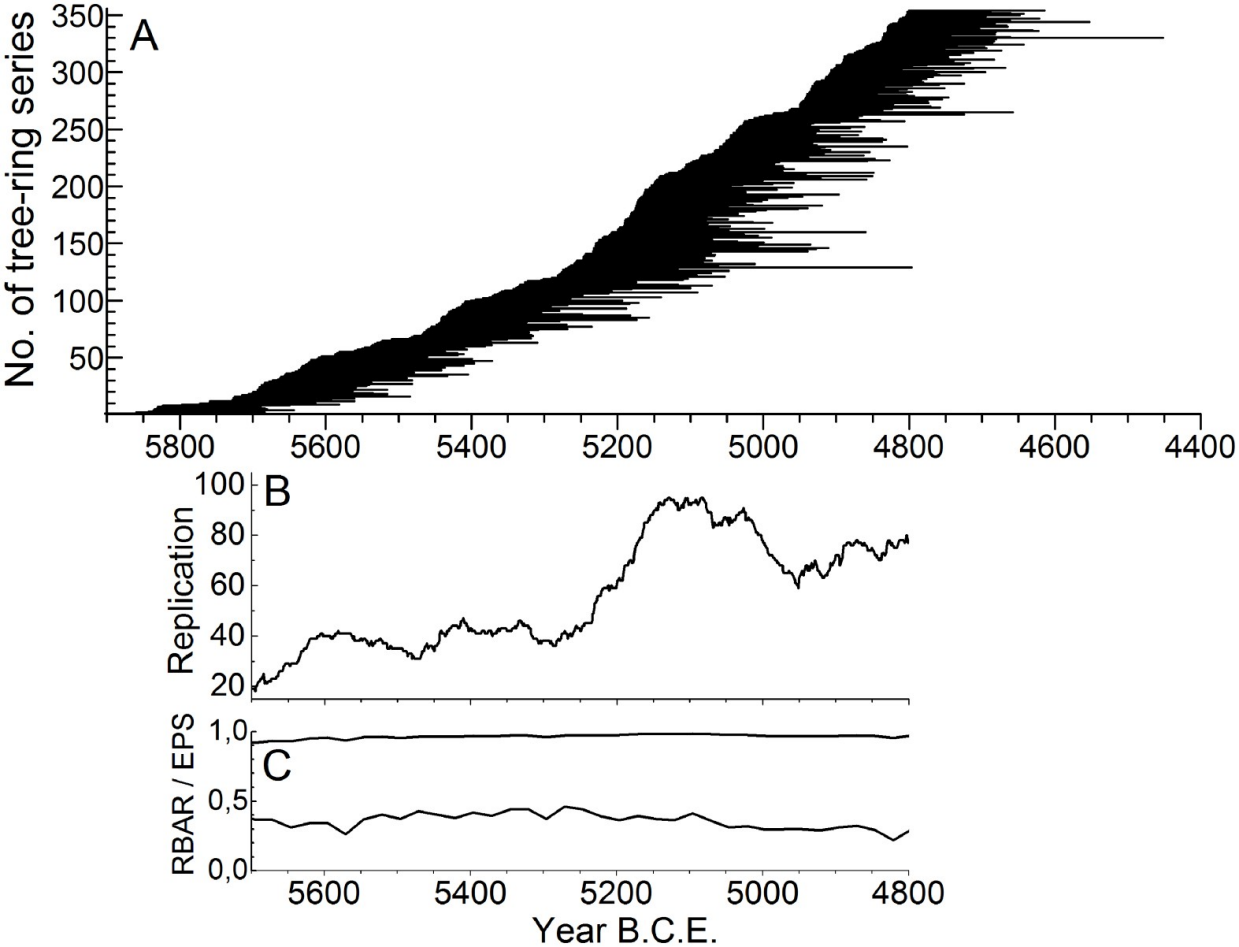
Tree-ring series used for precipitation reconstruction. (A) Lifespan of the oak trees from Main region, (B) replication (number of tree-ring series per year) and (C) inter-series correlation (RBAR) / Expressed Population Signal (EPS).

The tree-ring series used here lead to a high-quality TRW chronology spanning the period from 5700 to 4800 B.C.E. (Fig 5A–5C).

The TRW chronology consists of tree-ring series covering the entire LBK (Fig 5A). The mean length of the single series is 166.9 years, enabling us to capture high- to mid-frequency precipitation variability on regional scale.

In the first half of the investigated period (5700–5251 B.C.E.), the mean replication (40.9 tree-ring series per year) of the TRW chronology is considerably lower than in the second half (5250–4800 B.C.E., 76.0 tree-ring series per year). Thus, around 5280 B.C.E. the deposition frequency suddenly increases, leading to a high replication. This trend ceases 150 years later (5130 B.C.E.) before the replication decreases in the year 5030 B.C.E. while still maintaining a high level (Fig 5B).

Even when a change in replication is obvious, the values of EPS (mean = 0.96) and RBAR (mean = 0.36) are continuously high through the entire investigation period (Fig 5C). A well-replicated, high-quality TRW chronology could be constructed from this data set to reconstruct the hydroclimatic dynamic through the LBK.

### The evolution of climate between 5700 and 4800 B.C.E.

#### Spring-summer precipitation variability in southern Germany as reconstructed by tree rings

**Fig 6:**
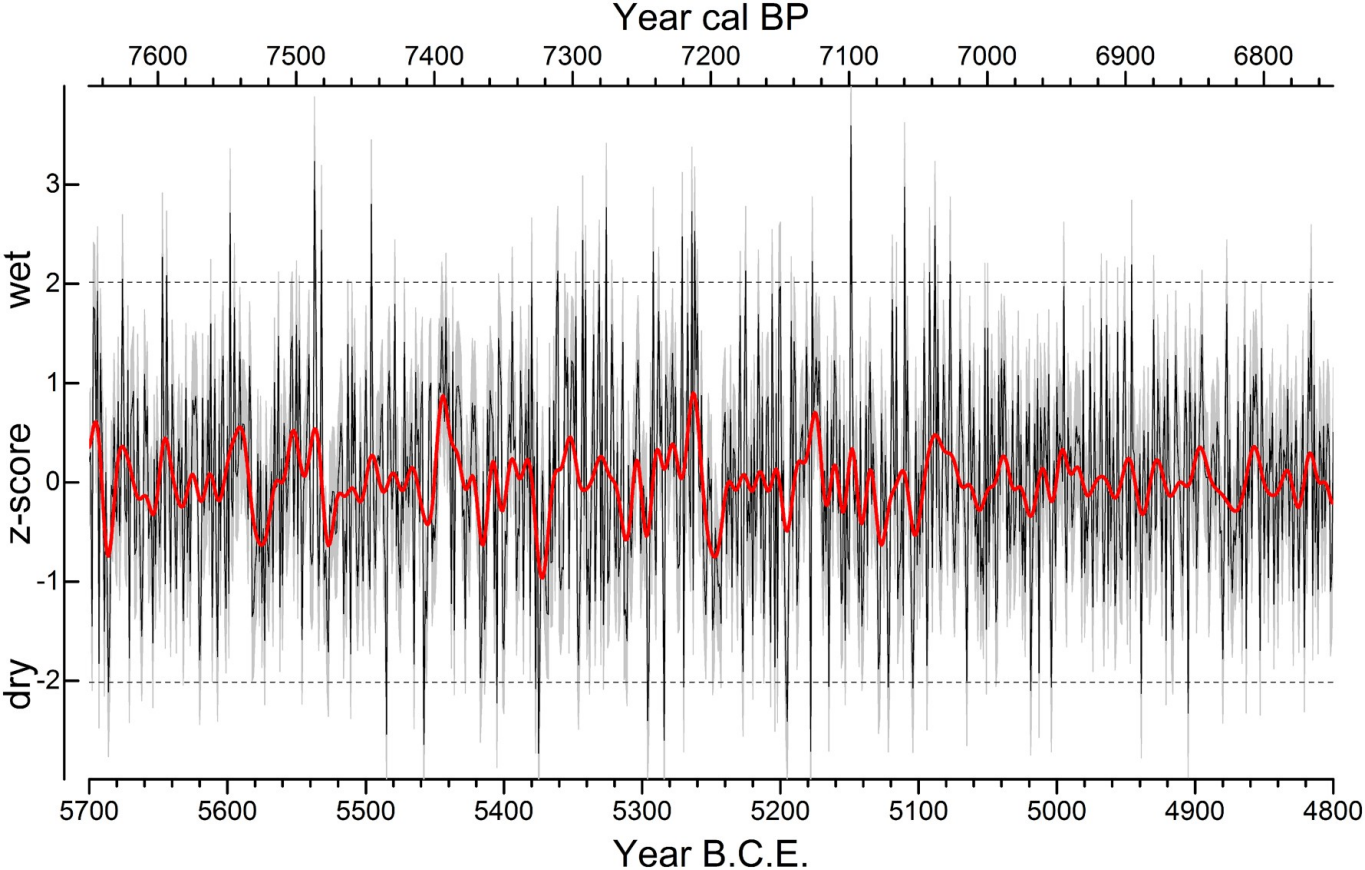
Dynamic of seasonal (black; Mean Absolute Error, MAE = grey shaded) and decadal (red) variability of spring-summer precipitation in southern Germany between 5700 and 4800 B.C.E. Dashed lines represent ±2 standard deviations.

Fig 6 shows the spring-summer precipitation variability [69] (DOI: 10.5281/zenodo.1326439) as depicted from tree-ring data during the LBK from 5700 to 4800 B.C.E. in southern Germany.

In Table 1, severe (<-2 / >+2 z-scores) droughts and wet periods during the 900-year period are listed.

**Table 1:** Severe (<-2 / >+2 z-scores) droughts and wet periods in southern Germany as reconstructed from tree rings.

Serial no.	Year B.C.E.	Drought (z-score)	Year B.C.E.	Wet (z-score)
1	5375	-2.73	5149	3.59
2	5178	-2.71	5537	3.23
3	5458	-2.64	5110	2.97
4	5284	-2.60	5496	2.80
5	5485	-2.54	5326	2.77
6	5195	-2.41	5264	2.73
7	5296	-2.40	5598	2.71
8	4905	-2.32	5088	2.58
9	5405	-2.22	5532	2.54
10	4939	-2.13	5262	2.53
11	5686	-2.11	5271	2.47
12	5019	-2.10	5343	2.44
13	5377	-2.08	5292	2.32
14	5104	-2.07	5647	2.27
15	5270	-2.06	5177	2.23
16	5122	-2.06	5077	2.23
17	5004	-2.06	4946	2.19
18	5165	-2.06	5361	2.13
19			5225	2.13
20			5092	2.12
21			5644	2.08
22			5676	2.05
23			5380	2.01

The precipitation reconstruction clearly shows that the period from 5700 to 5401 B.C.E. is characterised by four severe dry (5458, 5485, 5405, 5686 B.C.E.) and seven wet (5537, 5496, 5598, 5532, 5647, 5644, 5676 B.C.E.) seasons. The tree-ring reconstruction exhibits an almost two decade-long wet period (5448–5431 B.C.E.), the most pronounced in the entire LBK, as well as a second long-lasting wet phase (center year 5444 B.C.E.). A characteristic combination of a severe two- to three-year long drought followed by an extensive multi-year wet phase occurs at the transition of Late Mesolithic/Early Neolithic (5459–5431 B.C.E.). Such a typical pattern can be found quite often during the LBK.

The year-to-year variability of spring-summer precipitation remains high throughout the entire LBK, but is particularly distinct during the period from 5400–5101 B.C.E. In this 300-year long period, 10 severe dry (5375 (driest reconstructed season), 5178, 5284, 5195, 5296, 5377, 5104, 5270, 5122, 5165 B.C.E.) and 12 wet (5149 (wettest reconstructed season), 5110, 5326, 5264, 5262, 5271, 5343, 5292, 5177, 5361, 5225, 5380 B.C.E.) events could be detected (Table 1), demonstrating the high seasonal rainfall variation to which the LBK inhabitants were exposed. Taking into account that 18 severe dry (<-2 z-score) and 23 severe wet (>+2 z-score) seasons occurred throughout the entire investigated 900-year period, this demonstrates that most of the droughts/wet periods are detectable in Early Neolithic time. This 300-year period was hit not only by changing annual fluctuations, but also by several prolonged droughts and wet periods. At the beginning of Early Neolithic time, a multi-year period (center year 5372 B.C.E.) with below-average spring-summer precipitation occurred. The driest season (5375 B.C.E.) within the entire investigation period is part of this multi-year spring-summer drought. Six decades later, from 5313 to 5311 B.C.E., three consecutive dry seasons are evident from the tree-ring data. A half century later, a period of above-average precipitation was followed by below-average rainfall. This pattern of annual to decadal-scale hydroclimate variability lasts at least 25 years (5267–5243 B.C.E.). Thereafter, during roughly four decades, a period of a consistent hydroclimate occurred, with a distinct lack of severe droughts in the reconstruction (and only one very wet season). The next phase in a changing hydro-regime begins with a two-year persistent drought (5196–5195 B.C.E.) followed by a multi-year wet period (5177–5172 B.C.E.).

Within the later LBK, the year-to-year variability decreases and extreme dry/wet events become rare (5100–4801 B.C.E.). Nevertheless, four intensive dry (4905, 4939, 5019, 5004 B.C.E.) and four wet (5088, 5077, 4946, 5092 B.C.E.) seasons can be deduced from the tree rings. Consecutive dry/wet seasons are not apparent in this period. Thus, the transition from the young LBK into the Middle Neolithic can be characterised as a period with low year-to-year as well as low multi-year hydroclimate dynamic, at least when it is compared to the six previous centuries.

#### Evolution of temperature and solar irradiance in the LBK

**Fig 7:**
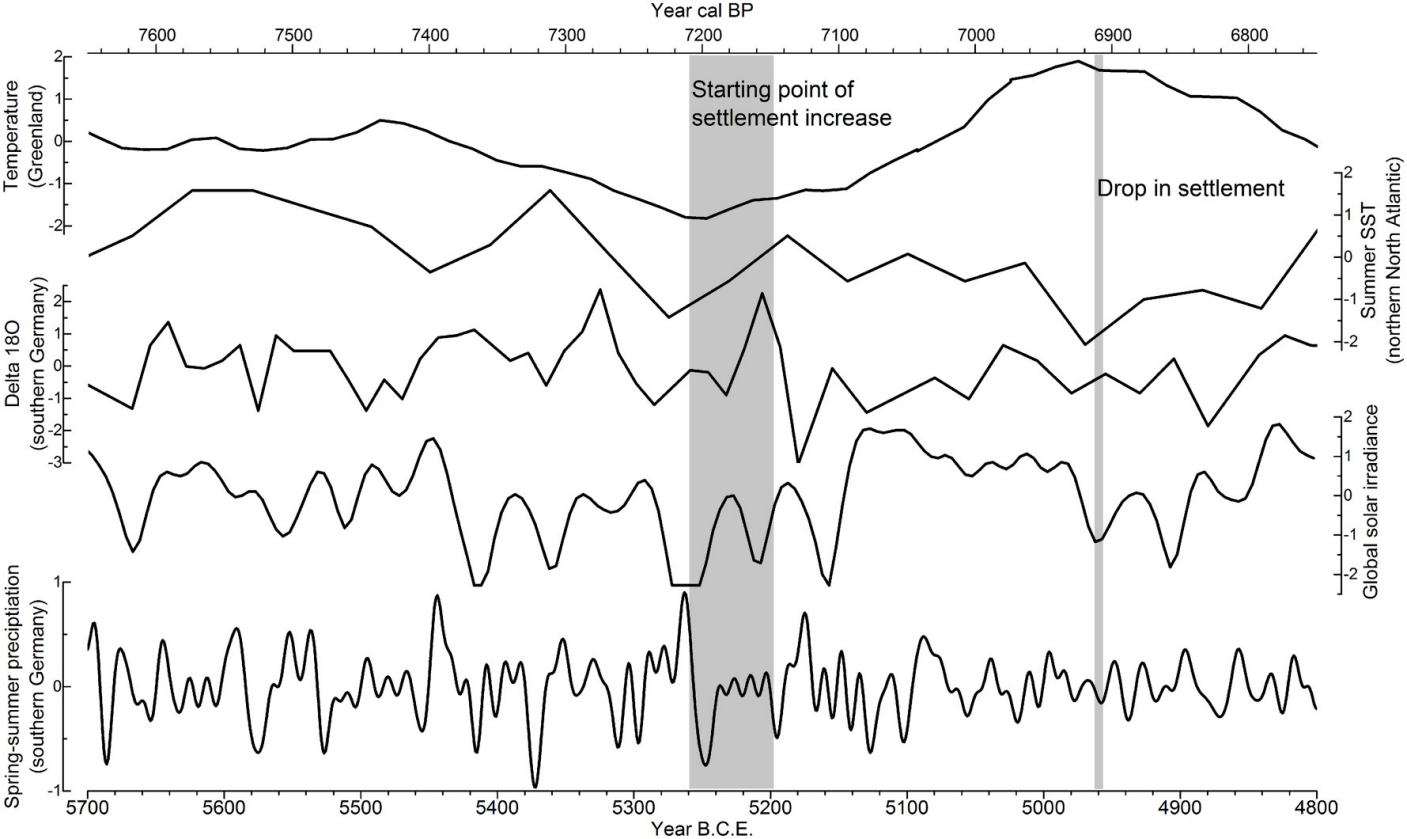
Climate variability during the LBK. Reconstructions of Greenland temperature [65], summer sea surface temperature in the northern North Atlantic [66], delta ^18^O in southern Germany [67], global solar irradiance [68] and precipitation (5-year lowpass filtered) for the Main region (southern Germany) from tree-ring data.

In Fig 7 the Greenland temperature variability from ice core data [65], summer sea surface temperature (sSST) in the northern North Atlantic [66], delta ^18^O in southern Germany [67], global solar irradiance [68] and the smoothed (5-year lowpass filter) precipitation variability in southern Germany is given.

As shown for the precipitation reconstruction, the variability of temperature, delta ^18^O and solar irradiance is also high during the investigated period. After a period of steady temperature until 5480 B.C.E. a continuous decrease in Greenland air temperature until 5250 B.C.E. occurs. After two to four decades of very low temperature (around 5255 B.C.E.), a steep rise occurred until 4975 B.C.E. First, the sSST followed the overall trend of the Greenland air temperature, but shows a continuous decline throughout the entire LBK, with a distinct drop around 5260 B.C.E. The delta ^18^O values (Ammersee, southern Germany) indicate a cooling event around 5280 and an even more pronounced cooling event around 5180 B.C.E. During 5420–5150 B.C.E. five periods of low global solar irradiance are likely as shown from the proxy data. A 20-year long period of very low solar irradiance appeared from 5260 to 5240 B.C.E. as well as from 5600 to 5300 B.C.E.

The period around 5250 B.C.E. is marked, from a climatic point of view, by distinct changes of all the above-mentioned reconstructions.

### Development of LBK settlement in Bavaria

While obvious differences in the settlement dynamics of nearby regions have been established for long, e.g. within different parts of Middle Franconia [30], the LBK is still often regarded as a quite homogeneous block. However, the results of the development of the LBK settlement presented here clearly show that there are in fact large differences, both between neighboring micro-regions and when viewed on a large scale.

#### Comparison of two micro-regions in southwest Bavaria

A study of the LBK-material from the Lech valley (Fig 1, no 6–7) generally revealed a very dynamic development of the settlement with three cycles of colonisation and depopulation during the older and younger LBK and during GG in the central and southern part of the study area (Fig 2). Despite the limited material basis, good concordance with the outcome of pollen analyses argue for the reliability of these results [28]. An interpretation of this result as repeated but not permanently successful attempts of the colonisation of this region is plausible.

A project carried out in the nearby region around the modern town of Höchstädt in the uppermost part of the Bavarian Danube valley [37] provided an opportunity to test these results (Fig 1, no 5). The first occupation occurred in the earliest LBK, followed by an amazing boom during the older LBK, when all nine sites were used simultaneously. Most sites were already abandoned in the middle LBK and only one site was continuously inhabited into the younger LBK (Fig 2). After a hiatus, resettlement of five sites is attested in GG. Compared to the first phase in the central Lech valley, settlement starts earlier, becomes much more intense and lasts longer in the Höchstädt region. The GG phase of settlement is also more intense than the third phase in the Lech valley. Hence, two of the three colonisation attempts in the central Lech valley took place at the peak of local population development in the neighboring Höchstädt region. The latter could therefore be the starting point of these population movements. The second colonisation wave in the central Lech valley, on the other hand, corresponds neither to a population maximum nor to a minimum in the Höchstädt region.

The mean annual precipitation totals differ substantially in the two study areas. While the precipitation sum is less than 750 mm per year in the Höchstädt region, it is between 850 mm and about 1000 in the Lech region (Fig 1. no 5 and 7). It is striking that the settlement in the area with the lower rainfall is characterised by an earlier onset, a longer duration of the settlement phases and a higher density of settlements (Figs 2 and 3)

#### General trend in Bavaria

In northern Bavaria, occupation starts very early, becomes quite intensive at the end of phase I, and remains continuously high thereafter. A smaller decline is only present at the beginning of local Middle Neolithic traditions. In southern Bavaria the onset of the first farming colonisation is delayed and staggered in different regions, the central Lech valley and the Isar estuary being latest. While in the large settlement areas in southeast Bavaria occupation seems continuous but uneasy, the smaller settlement zones in southwest Bavaria even show clear breaks.

The fact that a dense and continuous settlement is correlated with areas in which warm and dry climatic conditions prevail is also clearly confirmed in the Bavaria-wide analysis. In the colder and wetter regions, on the other hand, colonisation of the LBK is comparatively unstable or completely absent (Figs 1 and 3).

In contrast, it is hard to identify synchronous trends in supra-regional development (Fig 3). A phase of discontinuity seems present during the first half of the older LBK around 5260 B.C.E. From this time on, settlement increases and at least from 5200 B.C.E. on, an overall boom during the older and middle LBK is observed. The beginning of the younger LBK around 5060 B.C.E. is associated with disruptions in some regions (south-central Franconia) as well as with increases of settlement in other regions (central Lech and central Isar). The only general drop occurs at the end of the younger LBK around 4960 B.C.E., followed by a major hiatus. From about 4820 B.C.E. onwards, a wide-ranging resettlement by Middle Neolithic communities is seen.

## Discussion

### A possible link between hydroclimate and LBK settlement

The aim of this study is to assess possible links between temporal climate variability and changes of settlement during the LBK, similar to the well-attested dependence of the LBK settlement system on the spatial variability of climatic conditions. In both cases, the mechanism needs to be defined through which climate may have acted on the settlement system. The most probable route is the influence of the climate on agricultural production. Crop yield is determined by numerous factors: the nutrient supply of the cultivated soil, the characteristics of the cultivated species and varieties of crops, socio-economic parameters like the applied agricultural technology and the invested amount of labour, pests and finally climatic conditions being of particular importance [15,70–72]. Most of these factors might have been largely stable at least on a decennial scale during the LBK except the last two. Climatic variability is therefore regarded as a major factor causing annual deviations of Neolithic crop yield. Since summer crop cultivation is assumed to dominate the LBK, it must be mentioned that summer crops tend to be particularly sensitive to changes in precipitation rates [72]. Although significant fluctuation of spring-summer rainfall during the Early Neolithic has been proven, it is still unclear to which extent tree-ring width can directly be correlated to crop yield. The most important cultivated species were einkorn (*Triticum monococcum*), emmer (*Triticum dicoccum*), lentil (*Lens culinaris*) and pea (*Pisum sativum*) [15,22]. While cereals and lentils require sufficient water in spring, arid conditions in summer are in fact advantageous [73,74]. Pea, in contrast, requires a continuous water supply [74]. Hence, during early phases of the vegetation period, oaks and crops share the need for moist conditions, but in the later phases requirements diverge. Moreover, it is important to consider different growing regions: during the LBK, crops were typically grown on loess-soils covering well-drained gravel terraces. In contrast, the investigated trees originated from flood plain forests in reach of the groundwater level. Hence, crops might have been much more vulnerable to drought than trees from flood plain forests, which were still sensitive to drought. In addition, humidity could even cause diverging effects on trees and crops, depending on local conditions. Extremely humid conditions in summer could result in reduced crop yield but not in reduced tree growth. In this case, there seems to be only a partial correlation between tree-ring width and crop yield. However, as Bleicher [73] points out, under the warmer conditions of the Atlantic period, the correlation between the growth of trees and crops might have been close. However, tree rings are probably the best annual proxy for prehistoric crop yields currently available, even when they do not hold hydroclimatic information on a low-frequency scale.

Most striking is the observation that the consistency of settlement patterns over time is clearly dependent on the climatic characteristics of a region (Figs 1 and 3). Upper and especially Lower Franconia show much stronger continuity than all regions in southern Bavaria. While southern Bavaria on the whole is characterised by wetter and colder climatic conditions, some of the relatively advantitious regions like the Ries and the southeast settlement areas adjacent to the Danube and the Isar still show reasonably constant occupation. In contrast, particularly at the uppermost Danube and at the Lech, settlement fluctuates strongly. Hence, the stability of settlement is indubitably connected with the climatic conditions of a region. Archaeological data indicates permanent instability of the settlement system in southern Bavaria—an area that belongs to the least favourable settlement regions with respect to climatic conditions in the entire LBK, and the late date of colonisation might even be due to this [28]. Most likely, the economic system based primarily on only the four crop species einkorn, emmer, lentil and pea [22] reached its ecological limits. The combination of such a reduced variety of cultivated crops on the one hand with an extremely conservative economic strategy on the other hand probably caused the LBK-system to be highly vulnerable even to crop shortfall of just a single species. Though it is unclear to which extent adaptions of the crop plants occurred during the expansion of Neolithic culture, it seems plausible that the cultivated crops of originally Near Eastern provenance [15,75] were not perfectly adapted to lower temperatures and especially to higher summer humidity in Central Europe. These climatic factors may have resulted in reduced crop growth and increased risk, especially for fungal diseases [76,77]. Hence, it can be argued that agriculture, and by association the complete settlement system of the LBK in southern Bavaria, were highly sensitive and vulnerable to—positive as well as negative—climatic changes. For that, one might expect that stable environmental conditions enabling reliable adaption and planning of agricultural strategies would gain great importance. Low variability of climatic parameters would even allow for coping with phases of moderately reduced harvest or single years of crop loss, while highly unpredictable environmental conditions precluding successful planning and repeatedly causing crop loss might have serious negative consequences. Hence, a high frequency of extreme years will affect the agricultural system more than single dramatic climate events (e.g. severe summer drought). Concerning this, the very dynamic development of settlement observed during the older Neolithic especially in southern Bavaria could be interpreted as a result of very erratic environmental conditions. Despite this, with the exception of catastrophic events, the early farmers were most likely not harmed directly by slight changes in climatic conditions, but rather by the phenomenological response of crop plants to these.

### Comparing LBK settlement dynamics and hydroclimate

Archaeologically detectable changes in human settlement behavior are neither necessarily caused by changes in crop yield nor are changes in crop yield necessarily caused by climatic changes. Furthermore, human reactions to such changes often will be delayed in time. Nevertheless, it is plausible to assume large-scale and time-limited reasons in the case of phases of supra-regional and strong changes in the settlement system. Climatic changes are therefore considered in any case.

Overall, the comparison of the development in the different LBK settlement regions in Bavaria shows only weak synchronicity. If these tendencies are interpreted as an argument for a supra-regional development triggered by identifiable climatic events (Fig 6), one might expect long-lasting beneficial phases possibly during the expansion, and almost certainly during the settlement boom-phases in the 53rd– 52nd century B.C.E. as well as in the 48th century B.C.E. (Fig 7). Of course, the decline of the LBK around 4960 B.C.E. might be correlated with a climatic crisis, however, one needs to take into account that the apparently clear break between LBK and Middle Neolithic to some extent could also be an artefact of typological definitions. On the whole, however, heterogeneous settlement trends predominate over consonance, and therefore cast doubt on the relevance of a supra-regional mechanism of regulation of the population. In particular, the sometimes opposing developments in neighboring regions urge caution in interpretation.

The problem with the direct comparison of the data examined here is the temporal correlation: the climate data derived from tree rings have an annual resolution and are fixed in absolute chronological order for the year. On the other hand, the archaeological data have a resolution in the range of human generations and absolute chronological inaccuracies of some decades are possible. It should, however, be emphasised that the reconstructed spring-summer precipitation has a very high variability throughout the period from the oldest to the youngest LBK (Figs 6 and 7). This also applies to the above-mentioned periods of expansion and the supra-regional boom phase of the LBK. Remarkably, the time of the end of the LBK is characterised by clearly more even rainfall. Thus, no phases can be identified where there is a high probability of a direct relationship between the reconstructed precipitation on the one hand and the settlement dynamics on the other hand. Even with the inclusion of other climate proxies (Fig 7), this result remains. A simple comparison between climatically favourable or unfavourable phases and a clear reaction of settlement is therefore not possible.

### Early Neolithic coping with climate impacts

If at all, only a wide-ranging and synchronously occurring effect in the settlement dynamics could plausibly be explained by the impact of a single considerable climatic event. Surely, long-lasting or intense climatic events may actually have affected cultural systems relying mainly on agricultural production, but under the Holocene climatic regime in Central Europe, it is likely that only severe scenarios caused any significant impact. In fact, the archaeological data and the highly resolved precipitation reconstruction as well as the other climate data presented here does not allow for any definitive identification of single climatic events causing a response in Early Neolithic settlement.

In contrast, the presented data show that during the complete duration of the LBK, the climatic conditions were characterised by an extraordinarily high frequency of strong variations in spring-summer rainfall. Years with severe dry or wet seasons occurred erratically and within short intervals (especially until ~5100 B.C.E.). Most other phases of the Holocene Climatic Optimum were by far more balanced concerning spring and early summer rainfall, which is already true for the Middle Neolithic period. In fact, the Holocene Climatic Optimum was, by definition, the climax phase of the postglacial forest development in south-central Europe, but this should not be uncritically interpreted as an optimal phase for prehistoric agriculture as well. An enormous variability, at least of precipitation, is shown by tree rings. The increased mean temperature during the Atlantic period most likely led to an increase of extreme weather conditions such as heavy rainfall, storms and hail (e.g. [78]).

The economic system of the LBK had to cope with the challenges caused by the remarkably uneven agro-climatic conditions. In fact, the analysis of the settlement shows that the system was very successful and robust in climatically favourable landscapes: Apparently, sufficient surplus was generated to compensate for the presumed high frequency of crop losses due to high rainfall variability. Even in less climatically favourable areas, despite the permanent climate-related stress, successful economic management was possible at times. However, the system seems to be so unstable in such areas that even minor additional disturbances could cause a small-scale collapse of the population.

Remarkably, LBK farmers did not address this problem by attempting opportunistic adaptation to regional conditions and diversification and intensification of production. The Late Neolithic peasants of the 4th millennium B.C.E. in the circum alpine area were very successful, which however results in a completely different economic system and a decidedly mobile settlement dynamic [79–83]. This includes elements such as burning to keep the vegetation open as well as the increased use of hunting and gathering. In contrast, during the LBK a completely different approach was chosen, namely conspicuously conservative agricultural and overall cultural systems combined with a courageous colonial attitude [13,15] which might have been the most successful strategy. The desired ideal was evidently to maintain stationary settlements for centuries and to use the corresponding agricultural land permanently.

### Final remarks

Analysis of tree ring proxies is an excellent method for comparing archaeological and climatological developments: advantages include the high temporal resolution as well as the exact dating, as well as the close spatial connection to the archaeological settlement regions. In fact, the measured signal itself is a climate proxy for rainfall in spring and early summer, a crucial phase for the development of crop plants. The comparison of paleoclimatic data derived from tree rings and archaeological data, both from Bavaria, does not show indications for severe supra-regional cultural changes caused by single climatic events during the LBK including the transition to the Middle Neolithic. Thus, the long-term volatility of climatic conditions most likely influenced the formation of the overall conservative character of the entire LBK. An important reason of this development may have been the extremely high variability of precipitation and the associated difficulties in planning agricultural strategies.
